# Insulin-degrading enzyme is exported via an unconventional protein secretion pathway

**DOI:** 10.1186/1750-1326-4-4

**Published:** 2009-01-14

**Authors:** Ji Zhao, Lilin Li, Malcolm A Leissring

**Affiliations:** 1 Department of Molecular Therapeutics, The Scripps Research Institute, Scripps Florida, 5353 Parkside Dr., Jupiter, FL 32458, USA; 2 Department of Neuroscience, Mayo Clinic, 4500 San Pablo Road S., Jacksonville, FL 32224, USA

## Abstract

Insulin-degrading enzyme (IDE) is a ubiquitously expressed zinc-metalloprotease that degrades several pathophysiologically significant extracellular substrates, including insulin and the amyloid β-protein (Aβ), and accumulating evidence suggests that IDE dysfunction may be operative in both type 2 diabetes mellitus and Alzheimer disease (AD). Although IDE is well known to be secreted by a variety of cell types, the underlying trafficking pathway(s) remain poorly understood. To address this topic, we investigated the effects of known inhibitors or stimulators of protein secretion on the secretion of IDE from murine hepatocytes and HeLa cells. IDE secretion was found to be unaffected by the classical secretion inhibitors brefeldin A (BFA), monensin, or nocodazole, treatments that readily inhibited the secretion of α1-antitrypsin (AAT) overexpressed in the same cells. Using a novel cell-based Aβ-degradation assay, we show further that IDE secretion was similarly unaffected by multiple stimulators of protein secretion, including glyburide and 3'-O-(4-benzoyl)benzoyl-ATP (Bz-ATP). The calcium ionophore, A23187, increased extracellular IDE activity, but only under conditions that also elicited cytotoxicity. Our results provide the first biochemical evidence that IDE export is not dependent upon the classical secretion pathway, thereby identifying IDE as a novel member of the select class of unconventionally secreted proteins. Further elucidation of the mechanisms underlying IDE secretion, which would be facilitated by the assays described herein, promises to uncover processes that might be defective in disease or manipulated for therapeutic benefit.

## Results

Accumulating evidence from cell and animal modeling studies and human molecular genetics implicates impaired function of IDE in the pathogenesis of type 2 diabetes mellitus and Alzheimer disease (AD) [1-3]. IDE is the prototypical member of an evolutionarily distinct superfamily of zinc-metalloproteases possessing several features that distinguish it from conventional metalloproteases, including an "inverted" zinc-binding motif (HxxEH) [4] and an unusual tertiary structure [5-7]. Another distinguishing feature of IDE is its subcellular localization: the vast majority of IDE is present in the cytosol, with smaller amounts present in mitochondria, peroxisomes, and endosomes [8]. A small fraction of IDE–estimated to be 3% to 10% of the total–is also trafficked to the extracellular space, and it is this pool which interacts with known substrates of IDE, such as insulin and Aβ [9]. Despite a large number of studies demonstrating that IDE is secreted and/or associated with the cell surface (e.g., [10-13]), little else is known about the underlying export pathway(s).

In previous work, we showed that alternative translation initiation of IDE mRNA at an initiation codon upstream of the canonical one leads to the incorporation of a 41-amino acid N-terminal sequence [14]. Bioinformatic analysis showed that this sequence was highly conserved (e.g., ~80% identity between humans and pufferfish), implying that it serves some important function, and some prediction programs predicted it to be a *bona fide *signal sequence [14]. Subsequent experimental analysis, however, showed unequivocally that this sequence instead encodes a mitochondrial presequence, as demonstrated by the localization of green fluorescent protein tagged with this sequence, by electron microscopy, and by other methods [14,15]. Moreover, overexpression of IDE isoforms encoding this sequence had no effect on the degradation of extracellular substrates, in clear contradistinction to isoforms translated beginning at the canonical initiation codon [14,15].

**Figure 1:**
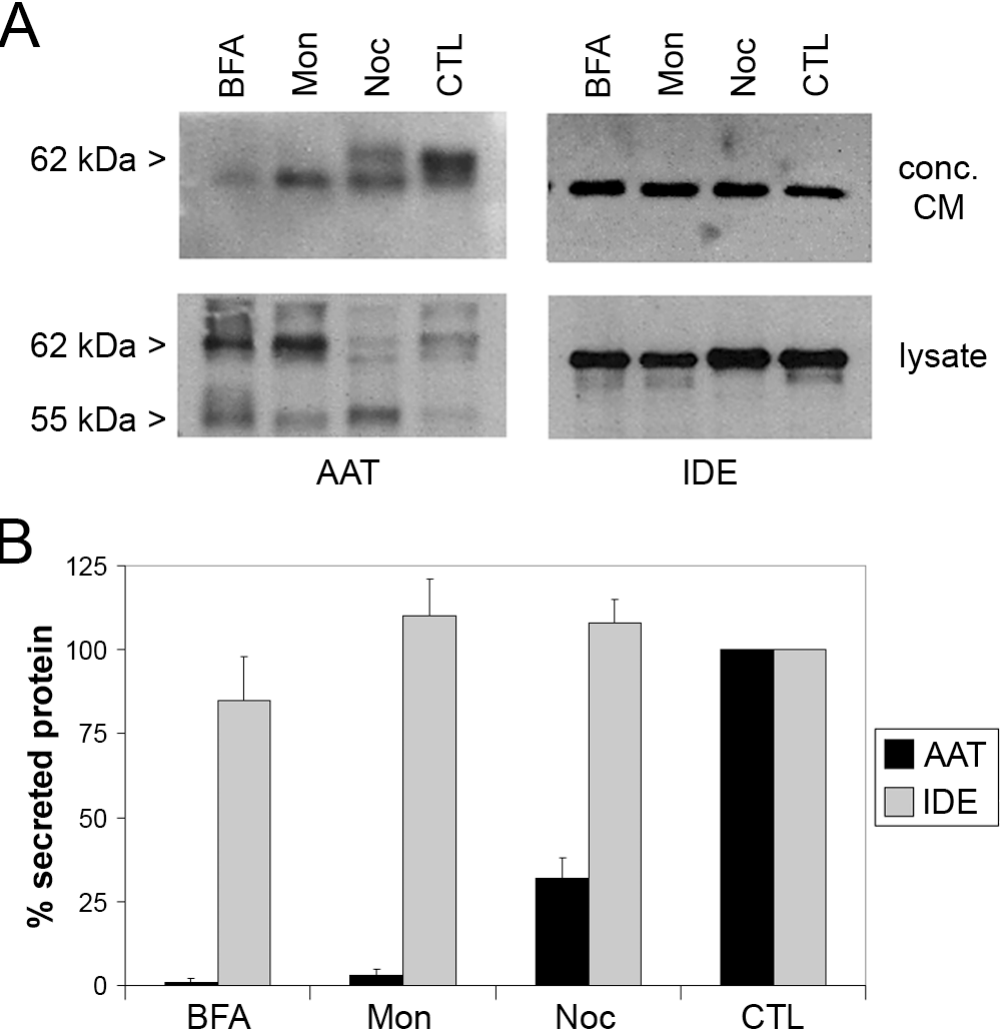
**IDE secretion is unaffected by treatments that block the classical secretion pathway**. ***A***, Representative western blots of concentrated conditioned medium (CM) and cell lysates from immortalized hepatocytes co-expressing IDE and AAT and treated with classical secretion inhibitors BFA (20 μM), monensin (Mon; 50 μM) or nocodazole (Noc; 3.3 μM) or vehicle only (CTL). Note that the mature, secreted form of AAT migrates at 62 kDa. ***B***, Quantification of western blots of secreted IDE and AAT. Data are mean ± SEM from 3 independent experiments.

Despite IDE's apparent lack of a classical signal peptide, it is possible that the protein may nevertheless be exported through the classical secretory pathway, whether mediated by undefined postranslational modifications (e.g., proteolysis to reveal a cryptic signal sequence), by association with heterologous classically secreted proteins, or by other means dependent upon a functional classical secretion pathway. To address this possibility, we co-transfected immortalized murine hepatocytes with full-length, unmodified cDNAs encoding human IDE and human α1-antitrypsin (AAT), a widely studied classically secreted protein [16]. Three days after transfection, cells were washed then treated with each of 3 different inhibitors of classical secretion: BFA (a blocker of ER-to-Golgi transport), monensin (an ionophore of monovalent cations that disrupts Golgi-dependent transport), and nocodazole (an inhibitor of microtubule polymerization that interferes with multiple forms of vesicular transport), or 0.1% EtOH as a vehicle control. Cell lysates and conditioned medium were collected, and the latter was concentrated 100-fold, then both were probed by western blotting for IDE and AAT. As expected, each of the treatments led to a significant decrease in the secretion of mature, ~62-kDa AAT and a concomitant increase in intracellular AAT or immature forms thereof (Fig. 1A, B). In striking contrast, however, the levels of secreted IDE were unaffected by any of these inhibitors of classical secretion (Fig. 1A, B). We confirmed that these compounds produced no significant cytotoxic effects at the concentrations tested, as judged by quantification of lactate dehydrogenase (LDH) release (data not shown).

**Figure 2:**
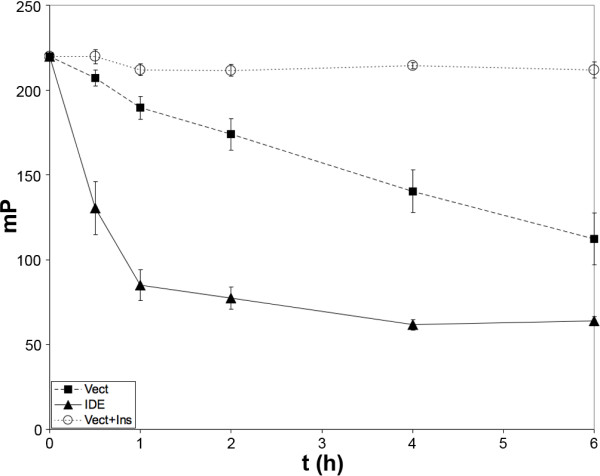
**A high-throughput-compatible fluorescence polarization-based assay for extracellular IDE activity detected by hydrolysis of FAβB**. Time course of the hydrolysis of FAβB (0.5 μM) in HeLa cells transfected 2 days previously with human IDE cDNA (IDE), empty vector (Vect) or the latter treated with insulin (20 μM; Vect+Ins) to inhibit extracellular IDE activity and carried out in 384-well format. Data are mean ± SEM for 16 wells/condition.

To study effects on secreted IDE proteolytic activity *per se*, we adapted our well-characterized fluorescence polarization-based Aβ degradation assay [17] for use in cells. To that end, HeLa cells transfected with IDE cDNA or empty vector were incubated for varying lengths of time with fluorescein-Aβ_1–40_-[Lys-LC-biotin] (FAβB) [17]. A time-dependent increase in FAβB hydrolysis was observed that was strongly increased in the IDE-overexpressing cells (Fig. 2). In agreement with prior findings [12], FAβB hydrolysis was inhibited nearly completely by insulin (20 μM), a competitive inhibitor of IDE. Collectively, these results suggest that nearly all the Aβ-degrading activity produced by these cells is attributable to IDE, making FAβB degradation a reliable surrogate marker of extracellular IDE activity.

**Figure 3:**
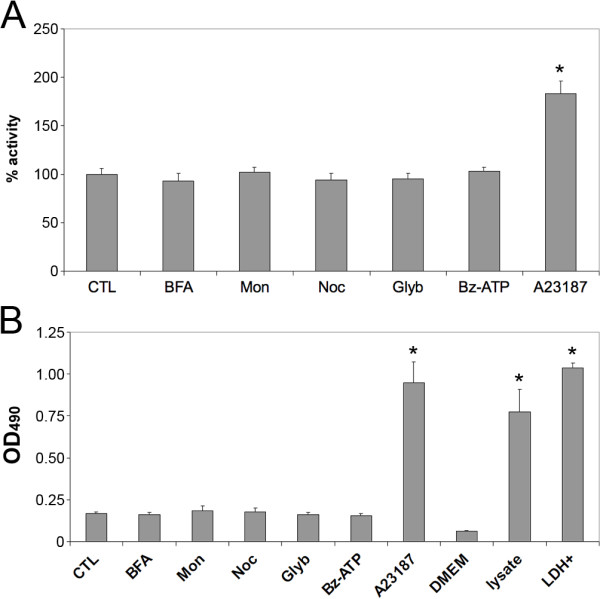
**Effects of inhibitors and stimulators of classical secretion on extracellular IDE activity and cytotoxicity**. ***A***, Extracellular IDE activity detected by FAβB hydrolysis in the absence (CTL) or presence of BFA (20 μM), monensin (Mon; 50 μM), nocodazole (Noc; 3.3 μM), glyburide (250 μM), Bz-ATP (100 μM), or A23187 (10 μM) in the presence of 1.8 mM Ca^2+ ^(A23187). Data are mean ± SEM for 16 wells per condition. *P < 0.01 by Student's T-test. ***B***, LDH release induced by treatment with the aforementioned chemicals, unconditioned medium (DMEM), HeLa cell lysate (lysate) or recombinant LDH (LDH+). Data are mean ± SEM for 4 wells per condition in 96-well format. *P < 0.01 by Student's T-test.

We then used this experimental paradigm to evaluate the effects of several inhibitors and activators of protein secretion on endogenously secreted IDE activity. Consistent with the results obtained above (Fig. 1), BFA, monensin and nocodazole at several concentrations produced no significant effects on the secretion of IDE from HeLa cells (Fig. 3A). We then tested several activators of conventional and unconventional protein secretion, including the ATP-binding cassette (ABC) transporter inhibitor glyburide, which is known to stimulate insulin secretion from pancreatic beta cells; Bz-ATP, a known stimulator of P2X7 receptors, which have been proposed to mediate the unconventional secretion of IL-1β [18]; and the calcium ionophore, A23187. No significant effect on IDE activity was elicited by glyburide or Bz-ATP, but significant enhancement was observed by treatment with A23187 for 2 h. Quantification of the effects of the different treatments on LDH release in parallel experiments, however, revealed that A23187 was significantly cytotoxic under the conditions tested (Fig. 3B). Subsequent analysis showed that treatment with A23187 for shorter durations (e.g. ≤ 30 min) failed to produce significant effects on extracellular IDE activity or LDH release (not shown).

The primary goal of the present study was to establish whether the secretion of IDE proceeds–directly or indirectly–via the classical secretion pathway. Using two independent methods, we show that the secretion of catalytically active IDE is not affected by multiple inhibitors of classical secretion. This result places IDE in the emerging category of unconventionally secreted proteins, which are characterized by the lack of a classical signal peptide, the lack of posttranslational modifications specific for the ER and Golgi compartments, and resistance to BFA and other inhibitors of Golgi-dependent export [19]. The list of proteins documented to be secreted by unconventional means is relatively small (i.e., approximately 20; see Ref. [19]), but it includes a number of medically relevant proteins, such as fibroblast growth factor-1 and -2 (FGF-2), interleukin-1α and IL-1β, thioredoxin, galectins, and certain heat-shock and cytoskeletal proteins [19,20]. Proteins associated specifically with neurodegenerative diseases have also been shown to be secreted by unconventional means, including α-synuclein [21] and prion protein [22].

The mechanisms underlying unconventional protein secretion are largely mysterious, and only beginning to be elucidated for just a subset of proteins. Nevertheless, unconventional secretion can be usefully subdivided into vesicular and nonvesicular pathways, with the former category applying predominantly to integral-membrane proteins, and the latter applying to soluble proteins [19]. As a soluble protein that lacks known ER/Golgi-specific posttransalational modifications, IDE would appear to be more likely to be secreted by nonvesicular means. Several different nonvesicular pathways have been proposed to underlie the export of different proteins, and our findings shed light on which might apply to IDE export. IL-1β, for example, has been proposed to be exported by activation of the purinergic ionotropic P2X7 receptor, resulting in release of the protein via microvesicle shedding [18]. The present study appears to exclude the possibility that IDE is secreted via the latter mechanism, since Bz-ATP, an activator of P2X7 receptors, was without effect (and HeLa cells have been shown to express the P2X7 receptor abundantly [23]). Secretion of FGF-2, on the other hand, has been shown to proceed by direct translocation across the plasma membrane, a process that is modulated by as-yet undefined cytosolic factors [24]. Whether IDE secretion is mediated by similar mechanisms as FGF-2 is currently unknown, but would appear to be a question worthy of future investigation. While the exact mechanisms underlying IDE secretion remain to be elucidated, one exciting aspect of unconventional protein transport is the possibility that protein-specific mechanisms may apply to the secretion of individual proteins. If this is true for IDE, it may be possible to selectively manipulate the secretion of IDE, without affecting the secretion of unrelated proteins.

An ancillary, more practical outcome of the present study is the demonstration that our well-characterized Aβ degradation assay [17] is suitable for use in cells and, when coupled with an LDH release assay, is capable of identifying false-positive cytotoxic compounds. Significantly, in HeLa cells and many other cell types (not shown), the vast majority of extracellular Aβ degrading activity is inhibitable by insulin, a highly selective IDE inhibitor, making this assay useful for detecting extracellular IDE activity directly. In the present study, the assay performed well at multiple scales, including 384-well format. Given the ratiometric nature of the fluorescence polarization read-out [17], this assay is well suited for high-throughput screening campaigns aimed at identifying chemical and genetic modulators of IDE secretion, which may in turn help uncover the detailed mechanisms underlying this poorly understood but biomedically important protein export pathway.

## Methods

### Chemicals

BFA, monensin, nocodazole, glyburide, Bz-ATP, and A23187 (Sigma) were prepared and stored according to manufacturer's recommendations.

### Cell Culture and Transfections

SV40-transformed wild-type murine hepatocytes were plated in 24-well plates (2 × 10^5 ^cells/well), transfected with appropriate cDNAs using TransIT LT1 according to manufacturer's recommendations (Mirus) and maintained in Hepatocyte Medium (3H Biomedical AB) supplemented with 20% fetal bovine serum. For experiments with HeLa cells a "reverse transfection" procedure was carried out in 384-well format (Mirus). Briefly, cDNA-TransIT LT1 complexes were prepared in Opti-MEM I low-serum medium (Invitrogen), added to 384-well plates (20 μL/well), then HeLa cells suspended in DMEM/20% FBS were added to the wells (20 μL/well; 10,000 cells/well). Cells were grown under normal cell culture conditions (37°C, 5% CO_2 _in a humidified chamber).

### Collection of Secreted and Intracellular IDE and AAT for Biochemical Detection

Three days after transfection, immortalized murine hepatocytes were washed 2 times in DMEM/0.1% bovine serum albumin (BSA), then incubated with the latter medium supplemented with appropriate chemicals or 0.1% EtOH as a vehicle control. Cells were pre-treated with compounds for 30 min, washed, then cells were allowed to condition the chemical-containing media for 2 h. Conditioned medium (250 uL/well, 6 wells/condition for each experiment) was collected, cleared of cellular debris by centrifugation, then concentrated 100-fold at 4°C using 2-mL YM-30 Microcon spin columns (Amicon) according to manufacturer's recommendations. Cells were washed twice with DMEM/0.1%BSA then lysed in PBS supplemented with 0.1% Triton X100 and 1× Complete Mini protease inhibitor cocktail (Roche) (30 uL/well, 6 wells/condition). After incubation on ice for 30 min, the lysates were centrifuged at 10,000 g at 4°C for 10 min to pellet cell debris and unlysed cells.

### Western Blot Analysis

Cell lysates and concentrated conditioned media were subjected to SDS-PAGE under reducing conditions using 10% polyacrylamide tris-glycine mini gels (Invitrogen) and transferred to nitrocellulose membranes as described [25]. Membranes were blocked in 5% non-fat milk in tris-buffered saline supplemented with 0.2% Tween-20 (TBS-T), incubated for 1 h with antibodies against IDE (IDE-1; 1:1000; Ref. [13]; generous gift of Dennis Selkoe, Harvard Medical School) or AAT (GAIT-80A; 1:1000; Immunology Consultants Laboratory), washed with TBS-T, probed with peroxidase-conjugated anti-rabbit IgG and anti-goat IgG secondary antibodies, respectively (1:5000; Vector Labs), and detected by enhanced chemoluminescence using SuperSignal West Pico Substrate (Pierce). Western blots were quantified using NIH Image 1.62 (NIH).

### Cell-based FAβB Degradation Assay

HeLa cells plated in 384-well format (10,000 cells/well) were washed twice in DMEM/0.1% BSA, then incubated at 37°C with the latter medium supplemented with FAβB (0.5 μM; 40 μL/well). Reactions were terminated by addition of DMEM/0.1% BSA supplemented with avidin (2.5 μM) and 1,10 phenanthroline (5 mM; 10 μL/well). Fluorescence polarization (λ_ex _= 485, λ_em _= 535) was determined using a SpectraMax M5 microplate reader (Molecular Devices). Controls included wells lacking cells (0% hydrolysis), wells supplemented with 10 nM recombinant IDE (100% hydrolysis) or excess insulin (20 μM) to establish IDE-dependent vs. IDE-independent hydrolysis. For experiments investigating the effects of chemicals on FAβB degradation, *in vitro *tests were conducted to guard against effects on the fluorescence polarization read-out or on the activity of recombinant IDE.

### Cytotoxicity assay

LDH release was quantified using the Cytotox 96 cytotoxicity assay (Promega) according to manufacturer's recommendations.

## Abbreviations

AAT: α1-antitrypsin; Aβ: amyloid β-protein; AD: Alzheimer disease; BFA: brefeldin A; Bz-ATP: 3'-O-(4-benzoyl)benzoyl-adenosine triphosphate; FAβB: fluorescein-Aβ_1–40_-[Lys-LC-biotin]; FGF-2: fibroblast growth factor-2; IDE: insulin-degrading enzyme; IL-1β: interleukin-1β; LDH: lactate dehydrogenase; P2X7 receptor: P2X purinegic receptor 7.

## Competing interests

The authors declare that they have no competing interests.

## Authors' contributions

JZ participated in the design of the study and executed the cell biological and enzymatic assays. LL generated the immortalized murine hepatocyte cell line, generated recombinant IDE and assisted JZ in the execution of all experiments. ML designed the experiments, analyzed data and wrote the manuscript.
